# EmoKbGAN: Emotion controlled response generation using Generative Adversarial Network for knowledge grounded conversation

**DOI:** 10.1371/journal.pone.0280458

**Published:** 2023-02-16

**Authors:** Deeksha Varshney, Asif Ekbal, Mrigank Tiwari, Ganesh Prasad Nagaraja

**Affiliations:** 1 Department of Computer Science & Engineering, Indian Institute of Technology Patna, Patna, India; 2 Samsung Research India, Bangalore, India; United International University, BANGLADESH

## Abstract

Neural open-domain dialogue systems often fail to engage humans in long-term interactions on popular topics such as sports, politics, fashion, and entertainment. However, to have more socially engaging conversations, we need to formulate strategies that consider emotion, relevant-facts, and user behaviour in multi-turn conversations. Establishing such engaging conversations using maximum likelihood estimation (MLE) based approaches often suffer from the problem of exposure bias. Since MLE loss evaluates the sentences at the word level, we focus on sentence-level judgment for our training purposes. In this paper, we present a method named *EmoKbGAN* for automatic response generation that makes use of the Generative Adversarial Network (GAN) in multiple-discriminator settings involving joint minimization of the losses provided by each attribute specific discriminator model (knowledge and emotion discriminator). Experimental results on two bechmark datasets i.e the Topical Chat and Document Grounded Conversation dataset yield that our proposed method significantly improves the overall performance over the baseline models in terms of both automated and human evaluation metrics, asserting that the model can generate fluent sentences with better control over emotion and content quality.

## Introduction

In recent years, there has been a significant rise in research interest in the domain of Conversational Artificial Intelligence (AI). The literature presented in [1, 2] proposed an end-to-end neural architecture based on the encoder-decoder framework for building conversational bots which can effectively mimic the real-life human conversations. However, these models often face the limitation of producing generic responses like “I don’t know”, “thank you”, “OK” etc. Conversational systems grounded on real-world knowledge can generate more semantically relevant responses. It is essential to associate the relevant topics, entities and relations referred in the user’s statement with real-world facts and diffuse them into the generated response. It becomes important to incorporate the related external knowledge extracted from Wikipedia, news and review articles as these are often not found in the structured databases. This is inspired by human cognitive skills of conversing with people. Unlike traditional dialogue systems, the goal of knowledge grounded conversation system is to provide the user with a highly proactive and informative conversation between the users.

In addition to grounding conversations with real-world information, it is equally important to capture user’s affective information in the form of sentiment and emotion to generate more fulfilling, intelligent and socially engaging responses. Understanding and acknowledging any feelings expressed in the user’s query could enable a dialogue agent to respond appropriately. A study on human-human interactions reports that humans interact with each other in a natural and social way [3, 4]. Hence, enabling the agent (goal-oriented or chit-chat bots) to understand human’s affects and/or empathy will further enhance its robustness and utility for any real-life applications.

Augmenting external knowledge to the conversational agent will make the conversation more realistic and exciting. Previous literature has mainly focused on augmenting the input of the Seq2Seq model by using the underlying knowledge associated with each utterance [5, 6]. Training using maximum likelihood (MLE) objective limits the model to generate high quality and contextually relevant responses. Although these models incorporate contents from the related knowledge base and take hints from the given emotion labels, they often fail to generate suitable responses as they are different from the ground-truth responses. Rather than just using these attributes (e.g. emotion, sentiment, personalization etc.) for generating the significant responses, they can be effectively used for evaluating the generated responses by providing useful references. However, this aspect has not been explored in these existing works. The Topical Chat dataset [7] contains coherent and engaging grounded conversations which are collected by providing the agents with appropriate external knowledge and emotion labels. The resulting dialogue lowers the gap between the generated response and ground truth response. Table 1 shows a snippet of such dialogue between the two agents from the dataset.

**Table 1:** A snippet of dialogue from the topical chat conversation.

	Utterance	Emotion
**Agent 1**	Did you know that a seahorse is the only fish to have a neck?	Curious
**Agent 2**	**Freshwater fish only drink water through the skin via Osmosis, Saltwater fish drink water through the mouth. Dolphins are friendly to human beings**.	Happy

Sentence used from the corresponding knowledge base is highlighted in bold.

The primary motivation behind our current work is to generate more socially engaging conversations, by considering emotion labels and relevant-facts associated with multi-turn conversations. The requirements of fluency and controllability are explicitly enforced by creating a model with two sub components, *viz*. a generator and two discriminators. The generator is in charge of generating knowledge grounded responses given a dialogue history and a knowledge base, while the two adversarial discriminators enforce emotional and knowledgeable response quality by trying to determine whether the item (dialogue history, emotion label, external knowledge, dialogue response) comes from the real data distribution. The system may simultaneously increase the quality of conversational responses and produce responses with varied emotions based on the target emotion label by teaching the generator in order to fool the discriminator.

In this paper, we present a novel, fully data-driven, knowledge grounded neural network based conversation model named as EmoKbGAN which utilizes both the underlying knowledge base and emotion labels to generate more contentful and engaging responses. We build upon the framework introduced by [8] and propose a multi-attribute discriminator training as a replacement of the MLE objective to supervise the training process. On the contrary, our method mainly contains two separate models: a transformer-based language model, which aims at generating pertinent responses with the support of attribute features provided as input to the model and the two discriminators guiding the generation process by calculating the probability of sampled sentences satisfying the given constraints.

We evaluate our proposed model on two benchmark datasets, *viz*. Topical Chat [7] and Document Grounded Conversation Dataset [9]. Experimental results show that our model is capable of generating contextually coherent and emotionally comforting responses. We find that generating both knowledge and emotion aware responses help make the conversations highly interactive. Both automatic and manual evaluation results show that our model considerably outperforms the baselines.

The key contributions and/or attributes of our current work are summarized as follows:

1. We propose a new framework, named as EmoKbGAN, based on the transformer architecture to generate long, informative, diversified, relevant and high-quality responses controlled by the varied emotions for knowledge supported conversations.
2. We utilize the theory behind training GAN on multiple-discriminators by simultaneously minimizing the loss signal provided by each discriminator (Knowledge and emotion discriminators).
3. We show with sufficient empirical analysis, through both automatic and human evaluation, that our proposed method generates more human-like and knowledge-aware responses.

## Related work

Traditional work on conversational Artificial Intelligence (AI) is broadly classified into the following two categories, *viz*. task-oriented and open-domain. Task-oriented bots intend to accomplish a specific task by having multiple interactions with humans, whereas the goal of open-domain bots is to have deep, engaging conversations with humans on all kinds of topics. To imitate human conversations, social chatbots must master various skills that humans possess naturally, such as retaining information from the outside world, reasoning based on conversational history, and building valid responses.

Rule-based dialogue systems [10] or template-based dialogue systems [11–13] are among the first techniques to be used to build the chatbots. These methods are time-consuming and do not scale up for different domains and applications. In recent times, with the rise of social networking platforms, a large amount of conversational data is open-sourced for research purposes. This has encouraged the establishment of retrieval-based [14–16] and neural-based [1, 17–19] methods for the data-driven conversations. Mostly, retrieval-based methods generate extremely fluent and syntactically correct responses as compared to neural generation based methods. However, the presence of an enormous repository of dialogue conversation between humans largely owes to the top-quality performance of the models. On the other hand, responses generated by neural generative models often lack in syntactic correctness but produce diverse and significant responses. Prior works provided approaches that can combine the qualities of retrieval-based methods with generative methods in the following works [20–23].

Besides, many new techniques, generative adversarial network (GAN) [24, 25] and conditional variational autoencoder (CVAE) [26–31] based models are also used to generate dialogue with a coherent structure. A new approach of considering sequence models as a reinforcement learning problem was introduced by [32, 33]. The model by [32] uses policy gradient methods to reward sequences that reflect three important conversational properties: informativity, coherence, and ease of answering by simulating dialogues between two virtual agents. An interesting work reported in [33] proposed a novel reward function which explores the implicit feedback via stance, sentiment, emotion, etc to optimize the future reward of a reinforcement learning-based neural conversation model. When training in an adversarial manner, a way to prevent manual determination of the reward functions using a discriminator is discussed [24]. To avoid producing repetitive responses, a discriminator based [25, 34] on a language model was implemented.

In recent times, there is an increasing interest to incorporate the external knowledge into the conversational agents. The authors in [35] proposed a benchmark task for intelligent open-domain dialogue generation grounded on relevant knowledge. They published a large dataset with conversations directly grounded with knowledge retrieved from Wikipedia. [36] use generative conversational networks to create conversational data automatically in order to train open domain social conversational agents and to take advantage of the wealth of language and knowledge data that is now available. [6, 37] uses the sequence to sequence approach for generating responses by conditioning them on both the conversation history and external facts. Apart from grounding conversations on the knowledge extracted from various Wikipedia and news articles, [38] uses profile information of users while generating conversations. They contain typical topics of human interest in user profiles that the speaker can bring up in conversation. [8] proposed an incremental encoder to encode multi-turn utterances as well as the knowledge in related documents along with a deliberation decoder to decode responses.

A hierarchical pointer network was proposed in [39] to attend and copy external knowledge hierarchically by the decoder in addition to the dialogue context. They showed improvement over the models proposed by [6, 38]. The authors in [5] used structured knowledge triplets to produce significant, and distinct responses using the technique of fact-matching and knowledge-diffusion. The Mem2Seq [40] network is known to effectively model the knowledge bases for conventional task-oriented dialogue systems. A multi-source seq2seq model is proposed in [41] which takes as input the current utterance and the N-best candidates along with a discriminator which can distinguish between human and machine-generated responses. [42] presents Prototype-KR, a novel knowledge selection approach, and Prototype-KRG, a knowledge-aware generative model, for neural response generation.

Monitoring the emotional contextual flow of a response can be handled in several ways. It can be controlled either by manually specifying the target emotion label [43–49] or by using a continuous representation of emotions [50]. These methods are evaluated by matching the generated emotion with a fixed target emotion. Text generation using variational autoencoders (VAEs) was presented by [46] to produce the sentences following a given sentiment or tense. In the same way, [51] presented a language model based on Recurrent Neural Network (RNNs) to generate the emotional sentences conditioned on their affect labels. Authors in [44] generated texts using emojis as the target labels by experimenting with several variants of conditional VAEs. Emotional chatting machine (ECM) [43] extended the seq2seq framework using three mechanisms, *viz*. emotion category embedding, internal emotion memory, and external memory to model the emotional factor of a sentence. EMOSen was proposed in [52] to utilize emotion, sentiment and multimodal information using an multimodal attention based conditional variational autoencoder (M-CVAE) framework. They conducted experiments on a large scale benchmark Sentiment Emotion aware Multimodal Dialogue (SEMD) dataset for the task of sentiment and emotion controlled dialogue generation.

The study in [49] added a lexicon-based attention mechanism to the Seq2Seq system, which generates responses by replacing terms with synonyms from an emotion lexicon. The research reported in [53, 54] introduced the stylistic transfer of the user behaviour, such as courteousness (e.g. polite, rude or neutral). PROSOCIALDIALOG [55] was introduced to respond to potentially unsafe user utterances by either ignoring or passively agreeing with them. They created a large-scale multi-turn dialogue dataset to teach conversational agents to respond to problematic content following social norms. Recently, Generative Adversarial Network (GAN) which uses discriminators as the guiding module has shown promising results for the task of text generation. SentiGAN, a novel controlled text generation framework by [45] proposes a model with multiple generators and one multi-class discriminator to generate texts with k types of sentiments. To enforce the generated samples to be sentiment compatible and realistic, they proposed a penalty based objective function in the generators. [48] performed controlled text generation using a reconstruction loss which alters between auto-encoding and back-translation loss functions and an adversarial loss to control attribute values such as indicative mood, past tense of a sentence. Recently, an adversarial empathetic dialogue system (EmpGAN) [56] has been proposed which uses a multi-resolution empathetic generator along with two interactive discriminators which extract the emotional factor of the sentences during dialogue generation. The work reported in [57] presents an adversarial model in which a sentiment-controlled dialogue generator is assisted by an adversarial discriminator which assesses the fluency and feasibility of the response generated from the dialogue history and a given sentiment label. Our work is different in the aspect that we consider more fine-grained labels for generating emotional responses as well as consider external knowledge for generating more engaging responses. Also instead of using a conditional variational generator [58] we employ a transformer-based generator which generates more powerful representations for texts.

Our present work situates itself within the data-driven paradigm of neural response generation along with controllable generative models conditioned on the emotional attributes as well as relevant facts. We extend the basic approaches by injecting side information from the background knowledge. We utilize the self-attention mechanism combined sequentially to form a sequential transformer model, instead of LSTM based model as a generator. Two discriminators are used to guide the training process along with adversarial learning. The experiments are performed on the knowledge-grounded Topical Chat dataset [7] and the Document Grounded Conversation dataset [9] containing a significant amount of human-human conversations in the open-domain setting. Our method tends to generate highly coherent and diversified responses.

## Methodology

In this section we first define the problem and then describe the proposed method.

### Problem statement

Our task is to generate emotion and knowledge controlled response for multi-turn conversations using relevant knowledge and emotion labels. Let *U* = *u*^(1)^, …, *u*^(*k*)^, …, *u*^(*K*)^ denote the set of K utterances of our multi-turn conversation. We represent m words of the *k*-th utterance as $u^{\left(k\right)}=u_{1}^{\left(k\right)},...,u_{i}^{\left(k\right)},\ldots ,u_{m}^{\left(k\right)}$. For every utterance *u*^(*k*)^, there is an external knowledge base $b^{\left(k\right)}=b_{1}^{\left(k\right)},\ldots ,b_{j}^{\left(k\right)},\ldots ,b_{n}^{\left(k\right)}$, where $b_{n}^{\left(k\right)}$ denote the *n*-th word in the *k*-th text based knowledge base. Every *k*-th utterance is given an emotion label *e*^(*k*)^ in the conversation. Hence, our task is to generate a response *y* given its associated knowledge base *b*^(*k*+1)^, emotion label *e*^(*k*+1)^ and the set of previous *k* knowledge-grounded utterances.

### Generator

**Fig 1:**
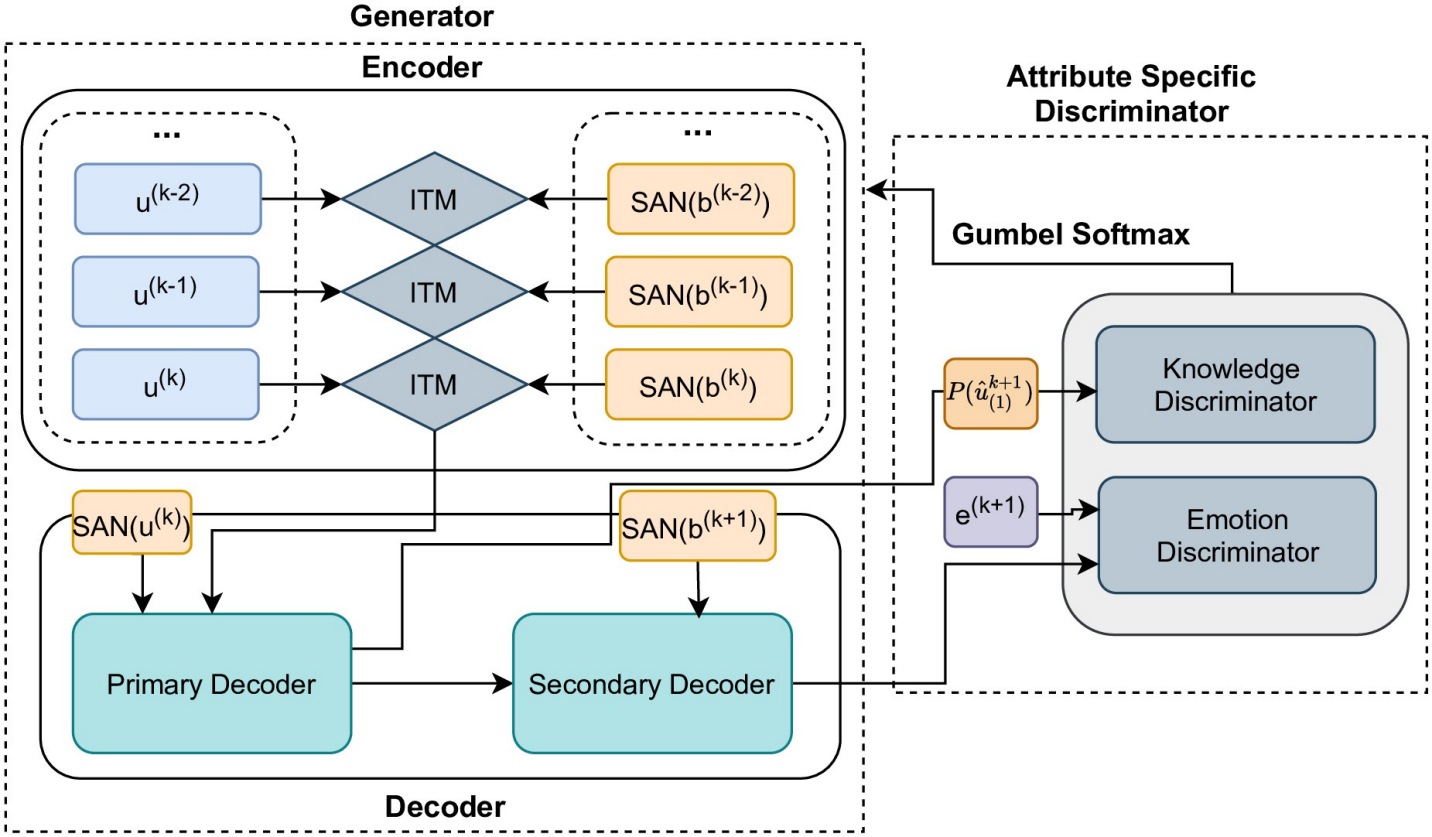
Proposed model architecture. ITM stands for Incremental Transformer Model, SAN stands for Self-Attention Network, variable ‘u’ stands for utterance and ‘b’ stands for knowledge bases.

**Fig 2:**
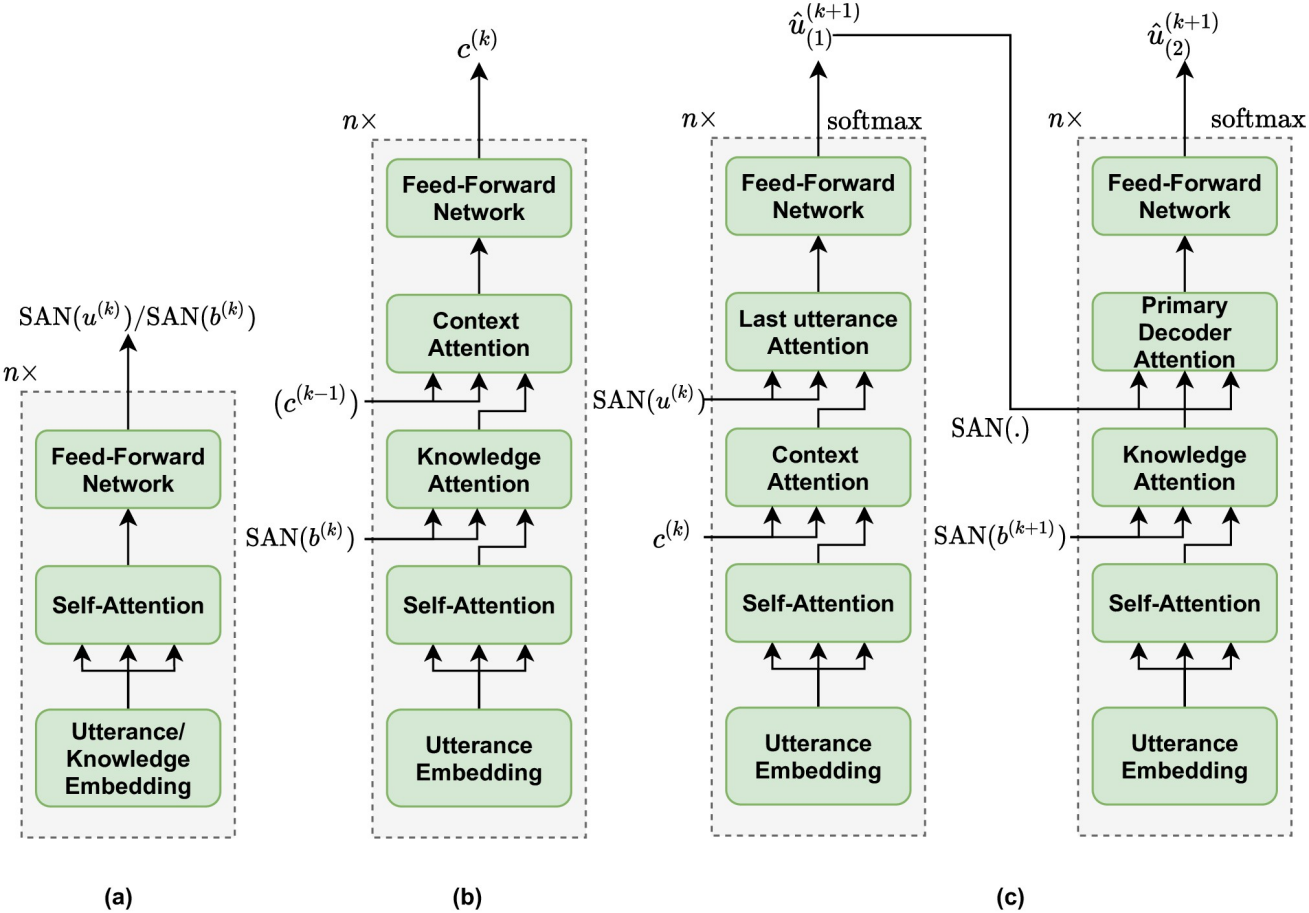
Detailed architecture of components in the EmoKbGAN model. (a) The Self-Attention Network(SAN). (b) Incremental Transformer Model (ITM). (c) Twin Decoder.

The real-world applications demand the conversational agents to be able to generate the output, which comprises of multiple sentences. For generating a multi-sentence response, we adapt the architecture by [8] which makes use of standard multi-head attention [59] to encode the utterances and knowledge base simultaneously. We employ a self-attention based generator capable of generating emotionally and contextually relevant responses for a conversation. The architecture is shown in Fig 1. and it consists of three key components:

- **Self-Attention Network**: The Self-Attention Network (SAN) leverages the positional self-attention mechanism by [59] to encode utterances and the knowledge associated with them for a given conversation.
- **Incremental Transformer Model**: The Incremental Transformer Model (ITM) takes *u*^(*k*)^ i.e the current utterance and *d*^(*k*)^ i.e corresponding knowledge base features as inputs. We then apply multi-head self-attention to compute an interdependent representation of the utterance and its related knowledge base.
- **Twin Decoder**: It is a multi-head self-attention based transformer decoder as described in [8]. The current utterance *u*^(*k*)^’s self-attentive representation and ITM’s output is fed as input to the primary decoder. The model depends on the context history information for response generation in the first step, whereas in the second step, we utilize the knowledge base *d*^(*k*+1)^’s representation which is associated with the target utterance *u*^(*k*+1)^. This allows utilizing the associated knowledge base for generating more quality responses Refer to Fig 2. for more details on the model components.

The encoder is an incremental transformer model which computes a knowledge as well as context aware representation of the utterance using the multi-head attention mechanism. It uses a self-attentive network (SAN) to encode the *k*-th utterance and knowledge.

$$\begin{array}{c} Ip_{u}^{k}=\left\{u_{1}^{\left(k\right)},\ldots ,u_{n}^{\left(k\right)}\right\} \end{array}$$ (1)

$$\begin{array}{c} A^{\left(n\right)}=MultiHeadAttn\left(Ip_{u}^{k},Ip_{u}^{k},Ip_{u}^{k}\right) \end{array}$$ (2)

$$\begin{array}{c} B^{\left(n\right)}=FFN\left(A^{\left(n\right)}\right) \end{array}$$ (3)

This process is denoted by *SAN*(.), for instance, *SAN*(*u*^(*k*)^) = *B*^(*n*)^, where n ranges from 1 to the number of identical layers in the self-attentive network. Also, the embedding of the current word and its positional encoding is used to obtain the representation of each word in the input utterance ${Ip_{u}}^{\left(k\right)}$.

The process of incremental encoding can be represented as $c^{\left(k\right)}=ITM\left(c^{\left(k-1\right)},B^{\left(n\right)},Ip_{u}^{\left(k\right)}\right)$. This module comprises of four components, *viz*. (i). first component is a multi-head self-attention to encode the current utterance *u*^(*k*)^; (ii). second component is a multi-head knowledge attention which is followed by multi-head context attention; and (iii). the last one is a position-wise fully connected feed-forward network.

$$\begin{array}{c} D^{\left(n\right)}=MultiHeadAttn\left(Ip_{u}^{k},Ip_{u}^{k},Ip_{u}^{k}\right) \end{array}$$ (4)

$$\begin{array}{c} E^{\left(n\right)}=MultiHeadAttn\left(D^{\left(n-1\right)},SAN\left(b^{\left(k\right)}\right),SAN\left(b^{\left(k\right)}\right)\right) \end{array}$$ (5)

$$\begin{array}{c} F^{\left(n\right)}=MultiHeadAttn\left(E^{\left(n\right)},c^{\left(k-1\right)},c^{\left(k-1\right)}\right) \end{array}$$ (6)

$$\begin{array}{c} G^{\left(n\right)}=FFN\left(F^{\left(n\right)}\right) \end{array}$$ (7)

$$\begin{array}{c} c^{\left(k\right)}=G^{\left(n\right)} \end{array}$$ (8)

The twin decoder consists of a primary and a secondary decoder. Similar to the encoder architecture, the primary decoder is also a sequence of several components. It starts with multi-head self-attention followed by multi-head context attention. The third component is a multi-head last utterance attention and the last one is a position-wise fully connected feed-forward network as before.

$$\begin{array}{c} Ip_{r}^{k+1}=u_{\leq i}^{\left(k+1\right)}=\left\{u_{0}^{k+1},u_{1}^{k+1},\ldots ,u_{i-1}^{k+1}\right\} \end{array}$$ (9)

$$\begin{array}{c} H_{1}^{\left(n\right)}=MultiHeadAttn\left(Ip_{r}^{k+1},Ip_{r}^{k+1},Ip_{r}^{k+1}\right)) \end{array}$$ (10)

$$\begin{array}{c} M_{1}^{\left(n\right)}=MultiHeadAttn\left(H_{1}^{\left(n\right)},c^{\left(k\right)},c^{\left(k\right)}\right) \end{array}$$ (11)

$$\begin{array}{c} P_{1}^{\left(n\right)}=MultiHeadAttn\left(M_{1}^{\left(n\right)},SAN\left(u^{\left(k\right)}\right),SAN\left(u^{\left(k\right)}\right)\right) \end{array}$$ (12)

$$\begin{array}{c} Q_{1}^{\left(n\right)}=FFN\left(P_{1}^{\left(n\right)}\right) \end{array}$$ (13)

The secondary decoder in contrast to the primary decoder has a multi-head knowledge-attention decoder followed by multihead attention on the outputs from the primary decoder.

$$\begin{array}{c} H_{2}^{\left(n\right)}=MultiHeadAttn\left(Ip_{r}^{k+1},Ip_{r}^{k+1},Ip_{r}^{k+1}\right)) \end{array}$$ (14)

$$\begin{array}{c} M_{2}^{\left(n\right)}=MultiHeadAttn\left(H_{2}^{\left(n\right)},b^{\left(k+1\right)},b^{\left(k+1\right)}\right) \end{array}$$ (15)

$$\begin{array}{c} P_{2}^{\left(n\right)}=MultiHeadAttn\left(M_{2}^{\left(n\right)},SAN\left({\hat{u}}_{\left(1\right)}^{k+1}\right)\right)),SAN\left({\hat{u}}_{\left(1\right)}^{k+1}\right))) \end{array}$$ (16)

$$\begin{array}{c} Q_{2}^{\left(n\right)}=FFN\left(P_{2}^{\left(n\right)}\right) \end{array}$$ (17)

The twin decoder works in two stages. The main force behind this is the information processing process by humans. In real-world human dialogues, people frequently write a preliminary draft of how to respond to the previous speech, then use background information to finish the answer or even offer questions. In the first stage, the decoder focuses on the contextual information provided by context utterances, whereas in the second stage, it utilizes relevant document knowledge to enhance the results obtained in the first stage eventually increasing the knowledge relativity and syntactical correctness in the generated responses. The twin decoder has the potential to generate appropriate sequence since it has extra information of knowing what the sequence to be generated might be by looking into the words decoded by the primary decoder [60].

After *n* layers, we use softmax to get the words probabilities decoded by primary and secondary decoder:

$$\begin{array}{c} P({\hat{u}}_{\left(1\right)}^{\left(k+1\right)})=softmax\left(Q_{1}^{\left(n\right)}\right) \end{array}$$ (18)

$$\begin{array}{c} P({\hat{u}}_{\left(2\right)}^{\left(k+1\right)})=softmax\left(Q_{2}^{\left(n\right)}\right) \end{array}$$ (19)

$$\begin{array}{c} L_{mle_{1}}=-\sum_{i=1}^{m}logP\left({\hat{u}}_{\left(1\right)}^{\left(k+1\right)}\right)) \end{array}$$ (20)

$$\begin{array}{c} L_{mle_{2}}=-\sum_{i=1}^{m}logP\left({\hat{u}}_{\left(2\right)}^{\left(k+1\right)}\right)) \end{array}$$ (21)

$$\begin{array}{c} L_{MLE}=L_{mle_{1}}+L_{mle_{2}} \end{array}$$ (22)

### Discriminator

Most of the existing GAN models use a binary classifier as the discriminator. We employ two attribute-specific discriminators to guide the training process. On one hand, there is a knowledge discriminator (KD) which is essentially a binary classifier that computes a binary probability distribution to distinguish the generated responses from the context as well as knowledge aware responses. Responses generated by the generation model containing necessary and adequate information in the interest of the conversation context seems more human-like, enabling the generator to incorporate more information from the external knowledge into the responses. We tend to model the interaction between the response, context, and knowledge base using the standard multi-head attention by [59]. As described in the previous section, we use the incremental transformer model with the primary decoder to obtain the context and knowledge aware response representation [8]. Lastly, we compute the probability *D*(*y*|*U*, *B*) that the response is human-like using Multi-layer Perception (MLP):

$$\begin{array}{c} D\left(y|U,B\right)=\sigma (MLP\left(P({\hat{u}}_{\left(1\right)}^{k+1})\right])) \end{array}$$ (23)

where *σ* is the sigmoid function.

On the other hand, our emotion discriminator (ED) is conditioned on some extra information such as emotion labels along with the generated response. In the discriminator, the response *u*^(*k*+1)^ and its correct emotion label *e*^(*k*+1)^ are combined in joint hidden representation, and fed as input to a single hidden layer of a Multi-layer Perceptron (MLP).

$$\begin{array}{c} D\left(y|U,E\right)=\sigma (MLP\left(\left[u^{\left(k+1\right)},e^{\left(k+1\right)}\right]\right)) \end{array}$$ (24)

where the bracket [·,·] denote concatenation and *σ* is the Sigmoid function.

Further, in order to train the model in an end-to-end manner using back-propagation, we use a Gumbel-softmax [61] distribution as a continuous approximation to sample the outputs from the generator. We start with a large value of *τ* and gradually anneal it to 0 as the training proceeds. Hence, the output of the generator eventually becomes a sequence of one-hot vectors which can be fed to the discriminator directly.

$$\begin{array}{c} y=softmax\left(1/\tau (h+g)\right) \end{array}$$ (25)

where the *g* are i.i.d samples from Gumbel(0,1) and *h* is the logits from the linear layer.

### Adversarial training

The objective of knowledge discriminator is to recognize if the response y comes from the generator or not. It computes the probability $D_{\phi_{1}}\left(y|U,B\right)$ that the response is knowledge as well as context aware given the context *U* and knowledge base *B* where *ϕ*_1_ denote the parameters of the first discriminator. On the other hand, the goal of the emotion discriminator is to generate responses conditioned on the target emotion labels. It computes the probability $D_{\phi_{2}}\left(y|U,E\right)$ that the response contains the desired emotion given the context *U* and the target emotion label *E* = *e*^(*k*+1)^, where *ϕ*_2_ denotes the parameters of the discriminator. Therefore, its objective function is to minimize the classification loss.

The loss of each discriminator $L_{D_{k}}$ is given by the following:

$$\begin{array}{c} L_{D_{k}}=-E_{y∼p_{data}\left(y\right)}\left[logD_{k}\left(y|U,A\right)\right]-E_{y∼G}[log\left(1-D_{k}\left(G\left(y|U,A\right)\right)\right] \end{array}$$ (26)

where A is the attribute, on which the utterances are conditioned on. However, the generator loss *L*_*G*_ is defined as the sum of the losses provided by each discriminator,

$$\begin{array}{c} L_{G}=-\sum E_{y∼G}\left[logD_{k}\left(G\left(y|U,A\right)\right)\right] \end{array}$$ (27)

where *D*_*k*_(*y*|*U*, *A*) and *G*(*y*|*U*, *A*) are the outputs from the both (k = 1 and k = 2) the discriminators and the generator, respectively.

Before starting training, we pre-train the generator on the training set {U,B} using the MLE objective. KD is pre-trained using context-knowledge aware responses as positive examples and responses produced by the pre-trained generator as negative examples. ED is trained on the texts with correct emotion categories as true examples and texts with incorrect emotion categories as the fake examples. Both the discriminator must learn to make predictions for the real and fake samples and the weights of the discriminator must be updated proportional to how correct or incorrect those predictions. The generator samples are fed to the discriminator which judges the sample as fake or real at adversarial training time. We collect true and fake samples using the topical chat and the isear dataset. The distribution of emotion classes present in the topical chat dataset are shown Table 2. To balance the number of emotion classes we drop some samples and add new samples from Isear dataset for the emotion class fearful, angry and disgusted. Fake samples were added by randomly assigning emotion classes to the dropped samples from the “Neutral” emotion class of the topical chat dataset. We added a total of 22792 number of fake samples which is equal to the number of true samples. The adversarial training is a min-max game played between the pre-trained generator and discriminator:

$$\begin{array}{c} \underset{G}{min}\underset{D}{max}\left(L_{G}\left(\theta \right)-L_{D_{k}}\left(\phi \right)\right) \end{array}$$ (28)

where the discriminator improves itself by learning to differentiate between the true and fake examples, while the generator upgrades itself by the signal provided by the discriminator on how well it managed to fool the discriminator. Fig 1. illustrates the structure of our proposed model.

**Table 2:** Distribution of emotion classes in topical chat dataset.

Emotion Classes	Original Count
Curious to dive deeper	101162
Surprised	38254
Disgusted	1848
Sad	3070
Neutral	51796
Happy	36845
Angry	1133
Fearful	1174

We summarize the steps for adversarial training in Algorithm 1.

**Algorithm 1** EmoKbGAN adversarial training

**Require**: The training set U, B

**Ensure**: The generator parameters *θ*

   The discriminator parameters *ϕ*_1_ and *ϕ*_2_

 1: Randomly initialize *θ*, *ϕ*_1_ and *ϕ*_2_

 2: Pre-train G using MLE objective.

 3: Produce responses using the pre-trained G;

 4: Pre-train KD using context-knowledge aware responses as positive examples and responses produced by the pre-trained generator as negative examples.

 5: Pre-train ED using texts with correct emotion category as true examples and texts with incorrect emotion category as fake examples.

 6: **for** epoch in number of epochs **do**

 7:  **for** *g* in g-steps **do**

 8:   Update *θ* according to Eq 27

 9:  **end for**

 10:  **for** *d* in d-steps **do**

 11:   Generate y from G as a negative example

 12:   Generate y from positive examples

 13:   Update *ϕ*_1_ and *ϕ*_2_ according to Eq 26

 14:  **end for**

 15: **end for**

 16: **return** *θ*, *ϕ*_1_, *ϕ*_2_

## Experiments

### Dataset

We evaluate our proposed model on knowledge grounded Topical Chat dataset with ∼11K human-human conversations [7]. The utterances in each conversation are grounded on eight broad topics, namely fashion, politics, books, sports, general entertainment, music, science & technology, and movies. Some of the utterances in the dataset may not have any knowledge associated with them, as they were written by the annotators with their own common sense knowledge. At each turn during a conversation, the utterances are annotated with the emotion it carries (e.g. angry, disgusted, fearful, sad, happy, surprised, curious to dive deeper, and neutral). The data is split into 5 distinct groups: Train, Valid Frequent, Valid Rare, Test Frequent, and Test Rare. Frequent set contains conversations on entities frequently seen in the training set. Rare set contains the conversations on entities those were infrequently seen in the training set. We show our experimental results on the frequent dataset.

We also perform experiments on Document Grounded Conversations [9] Dataset. The utterances are based on topics such as the title of the film, the cast, the introduction, the ratings, and some scenes. The average document length is about 200 words. We use a BERT-based emotion classifier that has been trained on the Topical chat dataset’s utterances to classify the target utterances of the CMU_DoG dataset. We used 200 sentences from the test set to assess our model’s results. On the test set, we received an overall accuracy score of 0.74.

Table 3 shows the sizes of the different groups of data. Our model is available at this link: https://github.com/deekshaVarshney/EmoKbGAN.

**Table 3:** Dataset details.

Topical Chat						CMU_DoG		
	Train	Valid Frequent	Valid Rare	Test Frequent	Test Rare	Train	Valid	Test
**#Conversation**	8628	539	539	539	539	3,373	229	619
**#Utterances**	188378	11681	11692	11760	11770	74,717	4,993	13,646
**Avg. # of Turns**	21.8	21.6	21.7	21.8	21.8	22.2	21.8	22.0

### Baselines

As per literature there is no work which jointly handles knowledge and emotion controlled dialogue generation for open-domain dialogue datasets. In order to prove the usefulness of our model we compare it with the following baselines:

1. **Seq2Seq**: This baseline is defined based on a simple encoder-decoder model [1, 17] with global attention [62]. The utterances and knowledge are concatenated and then fed as input to the model.
2. **Transformer**: This is a model based on multi-head attention [59] originally proposed for NMT. We concatenate the first three utterances and knowledge into a long sentence as its input.
3. **EmoKb-Seq2SeqGAN**: We implement a generative Seq2Seq model with global attention which takes both knowledge and context as input. We use two attribute specific discriminators for generating emotion and knowledge controlled responses.
4. **EmoKb-TransformerGAN**: We implement a transformer-based generator for modelling multi-turn open-domain dialogues with unstructured text facts along with two attribute specific discriminators.
5. **Incremental Transformer with Deliberation Decoder (ITDD)** [8]: It uses an incremental transformer-based model to encode utterances and documents, and a deliberation decoder to decode responses.
6. **GPT-2** [63]: It is a language model built on Transformer that was previously trained on Reddit dialogues, and it uses the input sequence to provide conditional probabilities for the output sequences. The GPT-2 model is used in its place as the generator, and both the dialogue and the knowledge information are passed to the model as input.
7. **DialogGPT**_***finetune***_ [64]: The model is built on the OpenAI’s GPT-2 architecture and was trained using 147 million Reddit chats. We start by creating a long text by concatenating all the conversation turns within a dialogue session. We swap out the generator of our proposed approach with the DialogGPT-2 model and feed it with both the conversation and the paragraph of knowledge.
8. **BERT** [65]: In this approach, contextual relationships between words (or subwords) in a text are learned through the employment of the Transformer’s multihead attention mechanism. The BERT model is used to replace the generator, and both the dialogue and the knowledge history are passed to the model as input.
9. **BART** [66]: This model comprises of a bidirectional encoder to encode the input sequences, and a left-to-right decoder which produces the appropriate response. We swap out the generator for the BART model and feed the model with both the conversation and the external information for our experiment.
10. **Emotional Chatting Machine** [43]: It models the emotional factor of a sentence using an extended version of the seq2seq paradigm, which includes three mechanisms: emotion category embedding, internal emotion memory, and external memory.
11. **EmpTransfo** [67]: It is a multi-head Transformer architecture with three feed-forward linear heads, responsible for generating the next emotion, utterance, and token.
12. **EMOSen** [52]: This baseline was proposed for sentiment and emotion aware multimodal dialogue generation. Since, we only have emotion and no multimodal information, we employ the Hierarchical Conditional Variational AutoEncoder (CVAE) with attention as proposed by the authors to replicate the model.

To prove the effectiveness of each module in EmoKbGAN, we also conduct the ablation studies for the multi-source generator and attribute specific discriminators. The models are *EmoKbG*: Incremental transformer with twin decoders; *KbGAN*: EmoKbG with only knowledge discriminator; and *EmoGAN*: EmoKbG with only emotion discriminator. To demonstrate the effectiveness of the twin decoder, we compare the results obtained from primary decoding and secondary decoding. The model name is *EmoKbGAN-SD*: EmoKbGAN without secondary decoder; *EmoKbGAN-PD*: EmoKbGAN without primary decoder.

### Experimental setup

We use the previous three utterances and their corresponding text-based knowledge as input. We use OpenNMT-py (https://github.com/OpenNMT/OpenNMT-py) [68] as the code framework. For all the models, the hidden size is set to 512. For our Seq2Seq based generator, we use a 3-layer bi-directional LSTM [69] with dot product attention. For transformer-based models, the number of encoder and decoder layers is set to 3. In multi-head attention, the number of attention heads is 8 and the filter size is 2048. We use the shared vocab and embeddings for the utterances, knowledge, and generated responses. Word embedding dimension is chosen as 512 empirically. The two networks, i.e. the discriminator and generator are trained alternately for about 200 epochs. For the generator, we use the ADAM optimizer whose learning rate is fixed to 0.0001. While decoding the responses we use beam search with beam size set to 5.

### Evaluation methods

#### Automatic evaluation

We use one of the most popular metrics for evaluating sequence like BLEU [70], perplexity (PPL) [1] and n-gram diversity (Div.) [7] to automatically evaluate the quality of generated responses.

1. **Perplexity**: We employed perplexity as an evaluation metric. Perplexity is defined by Eq 29. It is a measurement of how well a model can predict human responses. Generally, lower perplexity indicates better generation performance. Our various models are tested on the generation ability by computing perplexity on the validation data.
  $$\begin{array}{c} PPL=\text{exp}\{\frac{-1}{N}\sum_{i=1}^{N}log\left(p\left(y|U\right)\right)\} \end{array}$$ (29)
  where *N* is the total number of samples in the test set and *N*_*w*_ is the total number of tokens in the entire test set.
2. **BLEU**: We measure the significance of the generated responses by using BLEU, a word-based metric which measures n-gram overlaps with the gold response.
3. **N-gram diversity**: N-gram diversity is an automatic measure of the informativeness and diversity of sentences. We compute utterance level n-gram diversity where we find the unique number of n-grams divided by the total number of tokens in the generated sentence. We do that for every utterance and average it together to report the degree of diversity on the test data. The result is shown under the column Div. (n = 1) and Div. (n = 2) in Tables 4 and 5.

**Table 4:** Evaluation results using automatic and human evaluation metrics for baselines, ablation, and our proposed model on the Topical Chat Frequent dataset.

Models	PPL	BLEU%	Div. (n = 1)	Div. (n = 2)	Fluency	Adequacy	Emotional Content	Knowledge Relevance
Seq2Seq	80.2	0.73	0.91	0.87	1.22	0.35	0.25	0.22
Transformer	124.9	0.39	0.68	0.72	0.82	0.38	0.18	0.22
EmoKb-Seq2SeqGAN	83.3	0.56	0.92	0.86	1.19	0.29	0.25	0.28
EmoKb-TransformerGAN	98.1	0.37	0.70	0.75	0.95	0.39	0.25	0.29
ITDD	55.3	**1.04**	0.85	0.86	1.59	0.71	0.38	0.42
GPT-2	25.3	0.60	0.83	0.83	1.65	0.59	0.22	0.16
*DialogGPT*_*finetune*_	**20.4**	0.65	0.83	0.84	1.67	0.61	0.28	0.18
BERT	40.1	0.70	0.83	0.85	1.72	0.68	0.29	0.22
BART	38.2	0.79	0.82	0.85	1.76	0.69	0.26	0.20
ECM	89.4	0.45	0.85	0.86	1.42	0.65	0.29	0.30
EmpTransfo	28.3	0.80	0.87	0.88	1.85	0.76	0.48	0.32
EMOSen	67.9	0.50	0.80	0.83	1.65	0.53	0.19	0.17
**EmoKbGAN**	88.8	0.91	0.92	**0.89**	**1.87**	**1.17**	**0.52**	**0.55**
EmoKbG	97.8	0.90	0.92	0.88	1.50	1.08	0.40	0.49
KbGAN	105.8	0.63	**0.94**	0.86	1.52	1.11	0.40	0.50
EmoGAN	105.8	0.63	**0.94**	0.86	1.64	1.05	0.45	0.43
EmoKbGAN-SD	112.3	0.53	0.85	0.80	1.55	0.78	0.27	0.24
EmoKbGAN-PD	115.8	0.50	0.82	0.79	1.48	0.70	0.25	0.21

Bold face indicates leading results for each metric.

**Table 5:** Evaluation results using automatic and human evaluation metrics for baselines, ablation, and our proposed model on the CMU_DoG dataset.

Models	PPL	BLEU%	Div. (n = 1)	Div. (n = 2)	Fluency	Adequacy	Emotional Content	Knowledge Relevance
Seq2Seq	61.8	0.55	0.85	0.86	1.06	0.44	0.18	0.15
Transformer	80.3	0.39	0.70	0.74	0.90	0.50	0.18	0.17
EmoKb-Seq2SeqGAN	60.5	0.56	0.85	0.86	1.28	0.47	0.20	0.19
EmoKb-TransformerGAN	78.3	0.41	0.78	0.80	0.96	0.54	0.25	0.20
ITDD	37.1	1.08	0.89	0.89	1.68	0.92	0.30	0.54
GPT-2	22.3	0.59	0.81	0.82	1.70	0.63	0.24	0.17
*DialogGPT*_*finetune*_	**20.0**	0.70	0.81	0.83	1.71	0.65	0.25	0.19
BERT	39.3	0.71	0.85	0.86	1.79	0.76	0.28	0.22
BART	32.1	0.72	0.85	0.86	1.78	0.75	0.28	0.23
ECM	69.4	0.45	0.85	0.86	1.48	0.67	0.26	0.25
EmpTransfo	25.3	0.75	0.87	0.88	1.72	0.69	0.43	0.41
EMOSen	68.3	0.64	0.85	0.86	1.56	0.50	0.20	0.15
**EmoKbGAN**	38.5	**1.10**	0.94	**0.92**	**1.80**	**1.25**	**0.50**	**0.60**
EmoKbG	39.8	1.01	0.93	0.88	1.51	1.05	0.38	0.49
KbGAN	40.3	0.74	**0.96**	0.90	1.52	1.14	0.40	0.55
EmoGAN	40.8	0.75	0.95	0.91	1.64	1.08	0.48	0.43
EmoKbGAN-SD	51.3	0.63	0.86	0.81	1.51	0.57	0.32	0.22
EmoKbGAN-PD	50.8	0.60	0.85	0.81	1.49	0.52	0.30	0.20

Bold face indicates leading results for each metric.

#### Human evaluation

To measure the quality of the generated text from a human perspective [71, 72], we randomly sample 100 conversations from each model and with the help of ten well-trained experts with post-graduate exposure we evaluated the predicted responses using the following metrics:

1. **Fluency**: It measures the grammatical constructions.
2. **Adequacy**: It measures whether the generated response is contextually relevant.
3. **Knowledge Relevance** [5]: It checks whether the generated response contains all the relevant facts according to the ongoing conversation.
4. **Emotional Content**: It checks whether the generated response reflects the target emotion.

We measure the fluency, adequacy, and knowledge relevance on a 0-2 scale with ‘0’ signifying an imperfect or unfinished response, ‘1’ signifying a satisfactory response and ‘2’ signifying a correct response. We measure the emotional content on a scale of 0-1 with ‘0’ signifying the incorrect emotion and ‘1’ the correct emotion. To measure the agreement between two annotators, we compute the Fleiss’ kappa [73] score. We obtain a kappa score of 0.80, 0.86, 0.81, 0.72 for fluency, adequacy, emotional content, and knowledge relevance, respectively denoting “high agreement”.

## Results and analysis

We conduct the experiments using glove word embeddings for the input sequences. Tables 4 and 5 shows the summary of the performance for the baseline and the proposed models using both the automatic and manual evaluation metrics.

### Automatic evaluation results

In Tables 4 and 5, we observe that the proposed model has higher unigram and bigram diversities as compared to our baseline models *viz*. ITDD, EmpTransfo, ECM demonstrating that the models learn to decode lexically informative responses with great diversity on both the dataset. We observe relatively lesser repeated segments in the response generated by our proposed EmoKbGAN model owing to a good Div.(n = 1) and Div.(n = 2) score. Comparing our results to the baseline models, we see comparable BLEU score performance on the Topical Chat dataset. This could be explained by the fact that BLEU matches tokens from the predicted and the target responses using n-grams. However, there may be instances where the response is factually and contextually accurate but uses synonyms that do not exactly match the true response. On CMU_DoG, our proposed EmoKbGAN outperforms the baseline models on BLEU scores. Specifically, on comparing with EmoKb-Seq2SeqGAN and EmoKb-TransformerGAN, EmoKbGAN achieves a noticeable improvement on BLEU. This observation indicates that EmoKbGAN efficiently fuses the context and relevant knowledge base resulting in more diversified responses. We also observe an increase in PPL and decrease in BLEU scores when only the generator part of the model is used demonstrating the effectiveness of our attribute specific discriminators in the architecture. Our proposed EmoKbGAN, when compared to KbGAN and EmoGAN, although shows the comparable performance for the Distinct measure, it attains significant performance improvement for the BLEU and PPL metrics. This establishes the fact that the use of attribute specific discriminator in a joint manner enables the model to generate more grammatically correct responses than before. Additionally, we also observe a decrement in the scores of the EmoKbGAN-SD and EmoKbGAN-PD models when compared to the EmoKbGAN model. This shows the effectiveness of twin decoding in the decoder.

Apart from ITDD, EmpTransfo, ECM models, we also conduct experiments with the pre-trained language models such as GPT, DialogGPT, BERT, BART as described in the Baselines section. We replace the generator with the pretrained language models and pass the knowledge and utterances concatenated together as input into the model. We observe competitive performance by these models, however, our proposed EmoKbGAN outperforms these models by a huge margin on both the Topical Chat and CMU_DoG dataset. Pre-trained transformer models are language models that have already been trained on a huge amount of data. They require more labeled data and training time than uninitialized Transformer models since they are data-hungry. Overall, it appears that pre-trained language models can be used to ground open domain dialogues as long as we can locate a few dialogues carrying knowledge for fine-tuning. The baseline ITDD model and our suggested EmoKbGAN perform better than the pretrained baseline because ITDD and EmoKbGAN employ an incremental approach to combine the dialogue and knowledge information rather than just concatenating them. However, all metrics, with the exception of perplexity, show improved outcomes. This is because models learnt by fitting the same or a comparable distribution perform better on the measure because perplexity is calculated using actual data from test sets.

The original paper [7] proposed a transformer-based model, and observed a perplexity score of 33.8 and a Div.(n = 1) and Div.(n = 2) of 0.84 and 0.83, respectively. Following the diversity scores, we notice a significant improvement with Div.(n = 1) and Div.(n = 2) of 0.92 and 0.89, respectively, for our proposed model. However, it is to be noted that like us, they did not focus on emotion controlled dialogue generation. Moreover, our model does not filter any sentences as was done in [7], rather we take into consideration all the sentences from the knowledge base associated with a given utterance. We suppose that when we injected knowledge and used knowledge-aware and emotion-aware discriminators, the generated response contained knowledge words but had no sense of order which explains the increase in perplexity.

The ESTC dataset as proposed in [43] does not have utterances grounded in external information. It contains over four million real-world conversations obtained from Chinese micro-blogging, i.e., Weibo. The authors proposed the ECM model on the ESTC dataset. We perform experiment on the ESTC dataset using our transformer encoder and emotional discriminator model (TransEmoGAN) to show the effectiveness of our proposed GAN based approach. TransEmoGAN obtains BLEU of 0.54 and 0.60 on Topical Chat and CMU_DoG dataset whereas ECM obtains a BLEU score of only 0.32 and 0.28 on the Topical Chat and CMU_DoG dataset which we computed using the predictions obtained by implemneting the model available at this link: https://github.com/tuxchow/ecm. We observe that TransEmoGAN outperforms the ECM model with a huge margin on BLEU scores.

### Human evaluation results

We observe that all the models mostly produce comprehensible response and the models that ingest knowledge tend to produce better responses. Tables 4 and 5 illustrates that EmoKbGAN outperforms the other baseline models in terms of fluency, adequacy, emotion quality, and knowledge relevance on both the dataset. The increment in the fluency and adequacy scores with respect to baseline models verifies that the response generated by the proposed model comes out as more fluent and relevant. The emotional content score determines that the generated responses are more in line with the emotional sensitivity of the sentences. There also seems to be an improvement in the score of knowledge relevance, indicating an overall improvement in capturing the relevant facts from the associated knowledge base.

As mentioned in Baselines section, we also perform experiments using pretrained language models like GPT, DialogGPT, BERT, and BART apart from ITDD, ECM and EmpTransfo. On human evaluation also, we observe that even though these models exhibit competitive performance, our suggested approach *EmoKbGAN* exceeds them by a significant margin.

However, on comparing EmoKbGAN with the ablation models we observe that our model can aptly consume knowledge and emotion while generating substantially consistent responses. We observe that using the discriminators separately results in a similar performance. However, when combined we observe convergence and improvement in the overall performance for our proposed model. Hence, we justify the use of our attribute specific discriminators as we see an improvement in adequacy, emotional content, and knowledge relevance scores when compared with KbGAN and EmoGAN models. We may also note that the EmoKbGAN-SD and EmoKbGAN-PD models perform worse than our suggested method, EmoKbGAN. This demonstrates the decoder’s twin decoding function in action. Additionally, we perform extensive ablation study by varying the hidden size and number of layers in the model for the Topical chat dataset. The results are reported in Table 6. For hidden size 512 and number of layers as 3, we get the best results. When the number of layers is increased, we notice that overfitting occurs. We observed a decrease in the performance when we changed the hidden sizes. High-quality samples were created without employing MLE loss, however log-likelihood scores were quite low. When we combined MLE loss with the adversarial losses, we obtained lower perplexity, but did not observe any increment in the BLEU score. We also provide the ablation study by weighting the two discriminator losses. The results are reported in Table 7. When using different weights for discriminators, we do not observe much change in the BLEU and PPL scores.

**Table 6:** PPL and BLEU scores for varying number of layers and hidden sizes on topical chat test frequent set.

number of layers/hidden size	128	256	512	1024
1	129.3 / 0.76	118.6 / 0.75	89.6 / 0.86	134.6 / 0.87
2	120.1 / 0.78	118.1 / 0.76	89.1 / 0.88	130.1 / 0.88
3	118.8 / 0.79	116.8 / 0.81	**88.8** / **0.91**	132.8 / 0.90
4	119.0 / 0.78	117.0 / 0.78	89.0 / 0.88	132.0 / 0.89
5	124.2 / 0.81	118.2 / 0.72	90.2 / 0.81	128.2 / 0.87
6	125.7 / 0.71	119.7 / 0.68	90.7 / 0.81	131.7 / 0.86

**Table 7:** PPL and BLEU scores with weighted discriminator and MLE loss for the topical chat frequent test set.

	$\alpha .L_{D_{1}}+\beta .L_{D_{2}}$	*α*.*L*_*MLE*_ + *β*($L_{D_{1}}+L_{D_{2}}$)
*α* = 1 *β* = 1	88.8 / 0.91	55.6 / 0.89
*α* = 1 *β* = 2	89.3 / 0.90	56.1 / 0.91
*α* = 2 *β* = 1	90.8 / 0.88	57.8 / 0.90

**Fig 3:**
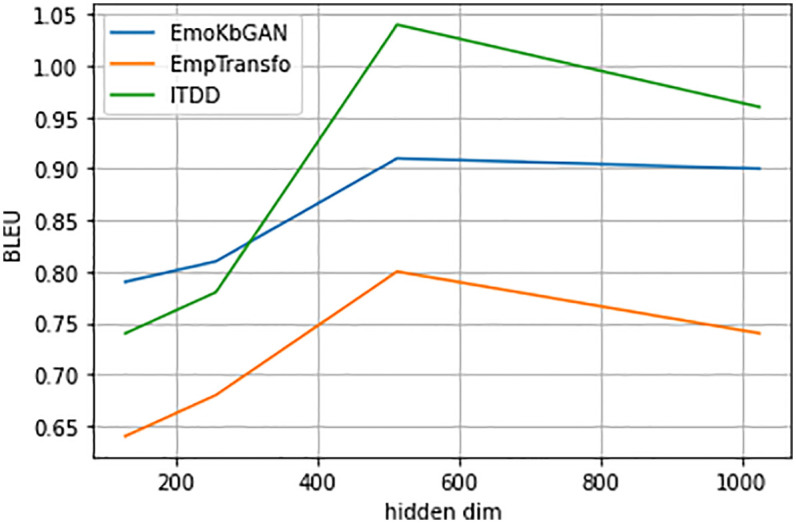
BLEU vs hidden size.

**Fig 4:**
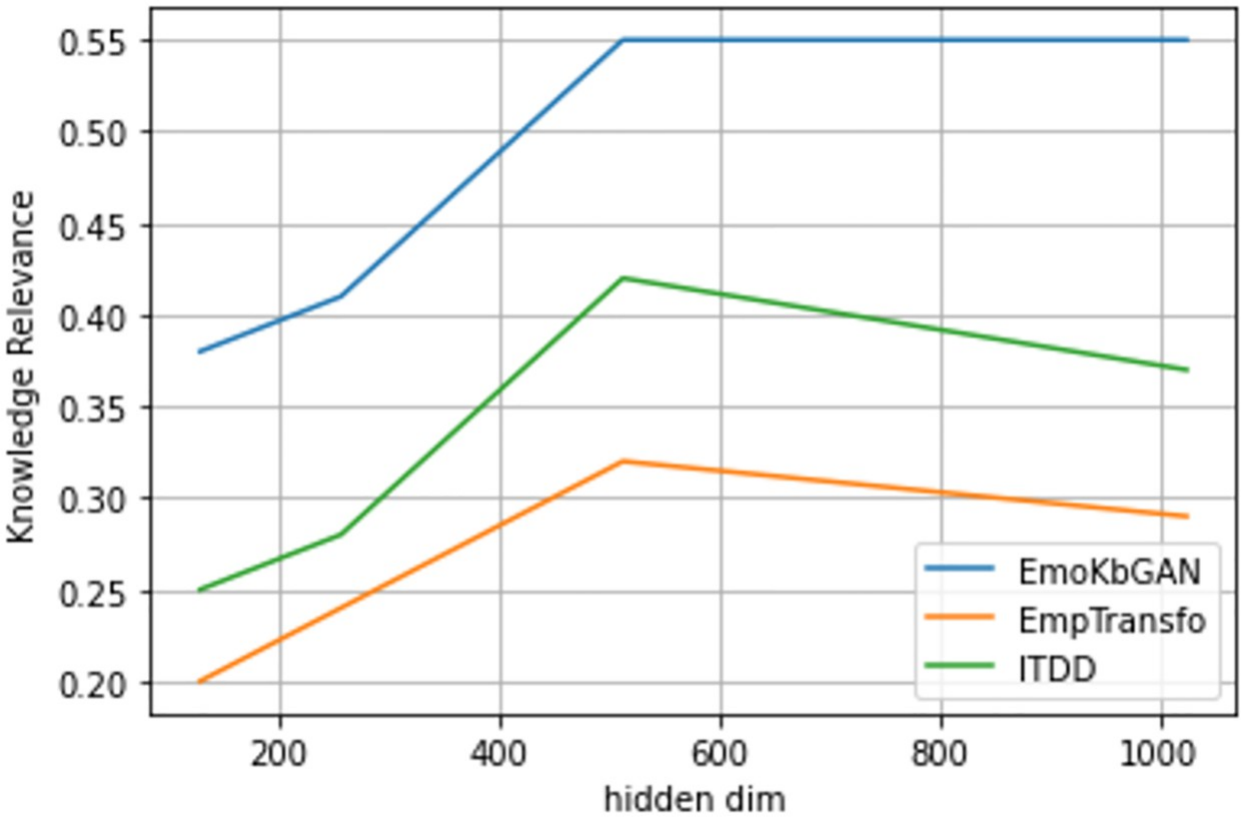
Knowledge relevance vs hidden size.

**Fig 5:**
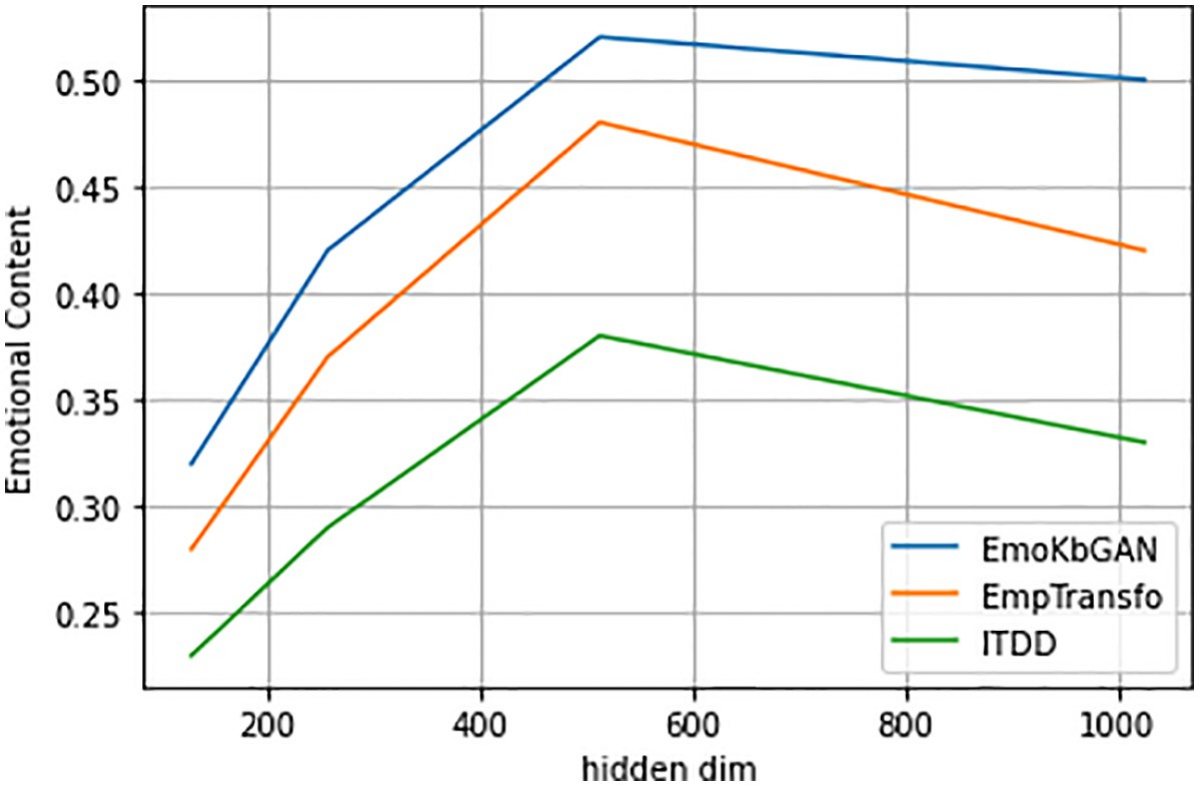
Emotional content vs hidden size.

In Figs 3–5, we show graph which plots the BLEU score, emotional content, and knowledge relevance of the frequent test set of topical chat vs. different model capacities (hidden size) given three encoder-decoder layers for different models viz. EmoKbGAN, ITDD and EmpTransfo. The figure clearly shows that with changing model sizes, our proposed model significantly improves in human evaluation and performs comparably in terms of BLEU scores.

### Statistical significance test

To compare every model, we performed a statistical significance test and carried out statistical hypothesis testing (t-test) [74], at 5% (0.05) significance level, which showed that the improvement in each model is statistically significant. We use the implementation provided at this link: https://github.com/rtmdrr/testSignificanceNLP to perform the t-test. By calculating the scores for each metric and for every sample in the test set, we will determine the significance of each individual metric (M) between two approaches A and B. The input consists of two files with the desired significance level and the output of each method (A and B) applied on the data X using metric M. We observe that performance improvement in the proposed model EmoKbGAN over the state-of-the-art, ITDD, EmpTransfo and other models on Diversity (Div. (n = 1) Div. (n = 2)) metrics is statistically significant using the Topical Chat Frequent dataset and BLEU and Diversity (Div. (n = 1) Div. (n = 2)) metrics of automatic evaluation using the CMU_DoG dataset and also on human evaluation metrics such as Fluency, Adequacy, Emotional Content, and Knowledge Relevance is significant with 95% confidence (i.e. p-value < 0.05) on both the dataset Topical Chat Frequent dataset and CMU_DoG.

### Detailed analysis

We further analyze the outputs generated from our proposed model to perform a detailed qualitative analysis of the responses.

#### Case study

Agent 1: *No, what happened in that game?*; Agent 2: *Georgia Tech defeated Cumberland but here’s the thing, they defeated them by a score of 222-0!*; Agent 3: *That is insane. How could that even happen?*; Target Response: *I don’t know but it did. It’s the highest scoring game in history. {Neutral}*; EmoKbGAN: *I think it could. Plus bowlers made twice as much as top football stars. {Neutral}*; EmoKb-Seq2SeqGAN: *I wonder how many people do that. {Surprised}*; EmoKb-TransformerGAN: *That would be a good way to get out of it. I wonder if it is the national by {Neutral}*.

In the example above, EmoKb-Seq2SeqGAN generates responses that are fluent, but inconsistent to the emotional context and knowledge, whereas EmoKb-TransformerGAN generates a response complementary to the context but emotionally relevant. We may note that our proposed model tends to generate emotionally and contextually relevant responses. Detailed examples with the associated knowledge base and emotion labels are shown in Tables 8 and 9. Also, it may be noted that in Table 8 the responses for our ablation model seems to align with the model architecture. The KbGAN model generates *“Good question. Did you know ESPN won an emmy for creating the yellow line?”* which is adequate as well as knowledge relevant response, whereas it fails to capture the relevant emotions in the generated response. On the other hand, EmoGAN model generates *“I think it would be fun to watch”* which is an emotionally relevant response.

**Table 8:** Generated examples from our baseline, ablation and proposed models on the test set.

Knowledge Base	
Football is a family of team sports that involve, to varying degrees, kicking a ball to score a goal. Unqualified, the word football is understood to refer to whichever form of football is the most popular in the regional context in which the word appears. Sports commonly called football in certain places include association football (known as soccer in some countries); gridiron football (specifically American football or Canadian football); Australian rules football; rugby football (either rugby league or rugby union); and Gaelic football. These different variations of football are known as football codes. the University of Iowa’s locker room for visiting football teams is completely painted pink in the 1960’s **top bowlers made twice as much as top football stars**—That the highest score ever in a football game occurred in 1916 when Georgia Tech defeated Cumberland 222-0 ESPN won an emmy for the creation of the superimposed yellow line representing the first down line for American football games. Former Partiots RB BenJarvus Green-Ellis has never fumbled the football in his NFL career.	
**Utterance 1**	No, what happened in that game?
**Utterance 2**	Georgia Tech defeated Cumberland but here’s the thing, they defeated them by a score of 222-0!
**Utterance 3**	That is insane. How could that even happen?
**Utterance 4**	I don’t know but it did. It’s the highest scoring game in history. **Emotion**: Neutral
**EmoKbGAN**	I think it could. **Plus bowlers made twice as much as top football stars. Emotion**: Neutral
**EmoKb-Seq2seqGAN**	I wonder how many people do that. **Emotion**: Surprised
**EmoKb-TransformerGAN**	That would be a good way to get out of it. I wonder if it is the national by **Emotion**: Neutral
**EmoKbG**	Well it was nice talking to you, have a good day! **Emotion**: Happy
**KbGAN**	Good question. Did you know ESPN won an emmy for creating the yellow line? **Emotion**: Curious to Dive deeper
**EmoGAN**	I think it would be fun to watch. **Emotion**: Neutral
Football is a family of team sports that involve, to varying degrees, kicking a ball to score a goal. Unqualified, the word football is understood to refer to whichever form of football is the most popular in the regional context in which the word appears. Sports commonly called football in certain places include association football (known as soccer in some countries); gridiron football (specifically American football or Canadian football); Australian rules football; rugby football (either rugby league or rugby union); and Gaelic football. These different variations of football are known as football codes. the University of Iowa’s locker room for visiting football teams is completely painted pink in the 1960’s top bowlers made twice as much as top football stars—That the highest score ever in a football game occurred in 1916 when Georgia Tech defeated Cumberland 222-0 ESPN won an emmy for the creation of the superimposed yellow line representing the first down line for American football games. Former Partiots RB BenJarvus Green-Ellis has never fumbled the football in his NFL career. The cat (Felis catus, or Felis silvestris catus, literally ““woodland cat””), often referred to as the domestic cat to distinguish from other felids and felines, is a small, typically furry, carnivorous mammal. It is often called house cat when kept as indoor pet or feral/feral domestic cat when wild. It is often valued by humans for companionship and for its ability to hunt vermin. There are more than seventy cat breeds recognized by various cat registries. **There are Cat Cafés in Japan and Taiwan where people can pay hourly to play with cats and have drinks!** when a cat reaches 15 years old it has usually spent 10 years of its life sleeping a physicist published his cat as a coauthor so he could write using first person plural without being criticized. the town of Talkeetna, Alaska has had a cat named Stubbs as its mayor since 1997. The cat in the Godfather 1 opening scene was a stray that was found on set. Failure to control the breeding of pet cats by spaying and neutering, as well as the abandonment of former household pets, has resulted in large numbers of feral cats worldwide, requiring population control. In certain areas outside cats’ native range, this has contributed, along with habitat destruction and other factors, to the extinction of many bird species. Cats have been known to extirpate a bird species within specific regions and may have contributed to the extinction of isolated island populations. Results of a genetic study in 2007 showed that all domestic cats descended from the Near Eastern wildcat and diverged around 8000 BC in the Middle East. A 2017 study confirmed that domestic cats are descendants of those first domesticated by farmers in the Near East around 9,000 years ago. As of a 2007 study, cats are the second-most popular pet in the U.S. by number of pets owned, after freshwater fish.	
**Utterance 1**	Do you like cats?
**Utterance 2**	Yeah, cats are cool. Do you own one?
**Utterance 3**	No, but many of my friends do have one or more, they sleep a lot
**Utterance 4**	Apparently, after living for 15 years, 10 of it is from sleeping. **Emotion**: Curious to dive deeper
**EmoKbGAN**	Do you have any pets? **We used to have a cat cafes in japan and Taiwan where people can pay hourly to play with cats and have drinks. Emotion**: Curious to dive deeper
**EmoKb-Seq2seqGAN**	Did you know there is 3g cell service on the internet. **Emotion**: Curious to dive deeper
**EmoKb-TransformerGAN**	I have not heard of it, but I do know that the internet has a lot of effort to sit around the levels of classics. **Emotion**: Surprised
**EmoKbG**	Yeah, they are usually good. **Emotion**: Neutral
**KbGAN**	True. Do you know much about cafes in Japan? **Emotion**: Curious to Dive deeper
**EmoGAN**	Would you rather have a cat? **Emotion**: Curious to Dive deeper

Sentence used from the corresponding knowledge base is highlighted in bold.

**Table 9:** Generated examples from our baseline, ablation and proposed models on the test set.

Knowledge Base	
Football is a family of team sports that involve, to varying degrees, kicking a ball to score a goal. Unqualified, the word football is understood to refer to whichever form of football is the most popular in the regional context in which the word appears. Sports commonly called football in certain places include association football (known as soccer in some countries); gridiron football (specifically American football or Canadian football); Australian rules football; rugby football (either rugby league or rugby union); and Gaelic football. These different variations of football are known as football codes. the University of Iowa’s locker room for visiting football teams is completely painted pink in the 1960’s top bowlers made twice as much as top football stars—That the highest score ever in a football game occurred in 1916 when Georgia Tech defeated Cumberland 222-0 ESPN won an emmy for the creation of the superimposed yellow line representing the first down line for American football games. Former Partiots RB BenJarvus Green-Ellis has never fumbled the football in his NFL career. Sports commonly called football in certain places include association football (known as soccer in some countries); gridiron football (specifically American football or Canadian football); Australian rules football; rugby football (either rugby league or rugby union); and Gaelic football. These different variations of football are known as football codes. There are a number of references to traditional, ancient, or prehistoric ball games played by indigenous peoples in many different parts of the world. Contemporary codes of football can be traced back to the codification of these games at English public schools during the nineteenth century. By the end of the nineteenth century, distinct regional codes were already developing: Gaelic football, for example, deliberately incorporated the rules of local traditional football games in order to maintain their heritage. During the twentieth century, several of the various kinds of football grew to become some of the most popular team sports in the world.	
**Utterance 1**	That is right! Hey do you like football?
**Utterance 2**	I do. I watched the superbowl a few weeks ago, kind of boring for the big game.
**Utterance 3**	Season is over!! when the season starts they only rest one weekend!
**Utterance 4**	Yeah, well apparently Iowa paints their locker room pink for visiting teams. **Emotion**: Curious to dive deeper
**EmoKbGAN**	That is so cute. They are very That too. **Emotion**: Happy
**EmoKb-Seq2seqGAN**	I agree. Do you like soccer? **Emotion**: Curious to dive deeper
**EmoKb-TransformerGAN**	That is a lot of football? I don’t know how much about you? **Emotion**: Curious to dive deeper
**EmoKbG**	It was so nice chatting with you as well. **Emotion**: Happy
**KbGAN**	I wonder if they did that **Emotion**: Surprised
**EmoGAN**	Yeah that would be true, and if the season 2 seasons are the best. Did you know that the NFL has only 11 minutes of live gameplay? **Emotion**: Curious to dive deeper
The cat (Felis catus, or Felis silvestris catus, literally “woodland cat””), often referred to as the domestic cat to distinguish from other felids and felines, is a small, typically furry, carnivorous mammal. It is often called house cat when kept as indoor pet or feral/feral domestic cat when wild. It is often valued by humans for companionship and for its ability to hunt vermin. There are more than seventy cat breeds recognized by various cat registries. There are Cat Cafés in Japan and Taiwan where people can pay hourly to play with cats and have drinks! when a cat reaches 15 years old it has usually spent 10 years of its life sleeping a physicist published his cat as a coauthor so he could write using first person plural without being criticized. the town of Talkeetna, Alaska has had a cat named Stubbs as its mayor since 1997. The cat in the Godfather 1 opening scene was a stray that was found on set. Failure to control the breeding of pet cats by spaying and neutering, as well as the abandonment of former household pets, has resulted in large numbers of feral cats worldwide, requiring population control. In certain areas outside cats’ native range, this has contributed, along with habitat destruction and other factors, to the extinction of many bird species. Cats have been known to extirpate a bird species within specific regions and may have contributed to the extinction of isolated island populations. Results of a genetic study in 2007 showed that all domestic cats descended from the Near Eastern wildcat and diverged around 8000 BC in the Middle East. A 2017 study confirmed that domestic cats are descendants of those first domesticated by farmers in the Near East around 9,000 years ago. As of a 2007 study, cats are the second-most popular pet in the U.S. by number of pets owned, after freshwater fish.	
**Utterance 1**	What? That is crazy! lol There is a cat named Stubbs who is the mayor of a town called Talkeetna in Alaska! How does that work? lol.
**Utterance 2**	I wonder who that cat ran against? It would’ve been funny if the other candidate was a dog!
**Utterance 3**	Hahah Yes it would be! I’ve had a good time chatting with you!
**Utterance 4**	Me too. Have a great day! **Emotion**: Happy
**EmoKbGAN**	Have a great evening! **Emotion**: Happy
**EmoKb-Seq2seqGAN**	You too! **Emotion**: Neutral
**EmoKb-TransformerGAN**	You too! **Emotion**: Neutral
**EmoKbG**	Great chatting with you as well! Have a great day! **Emotion**: Happy
**KbGAN**	Have a great evening! **Emotion**: Happy
**EmoGAN**	Ha! Well I got to go, nice chatting with you. Have a great night! **Emotion**: Happy

Sentence used from the corresponding knowledge base is highlighted in bold.

Also, as seen in Table 2, we observe that the imbalanced emotion class distribution in the topical chat dataset results in low performance of our EmoGAN model which, in turn, affects the quality of responses generated by our proposed model. The classes “*Curious to dive deeper*”, “*Surprised*”, “*Neutral*”, “*Happy*” are well-classified by our emotion discriminator thereby producing more responses which begin with words like “*Did you know that?*” and “*I wonder*”. Whenever the agent wants to terminate the conversation, our proposed model generates fluent and adequate sentences. For example, Agent 1: *What? That is crazy! lol There is a cat named Stubbs who is the mayor of a town called Talkeetna in Alaska! How does that work? lol*; Agent 2: *I wonder who that cat ran against? It would’ve been funny if the other candidate was a dog!*; Agent 3: *Hahah Yes it would be! I’ve had a good time chatting with you!*; Predicted Response: *Have a great evening!*; Target Response: *Me too. Have a great day!*. Also as seen in Table 9, in the second example, the ablation model also generates adequate responses.

There are also some cases where EmoKbGAN does not generate the desired responses. For example, there exist many responses with short texts, such as “*I didn’t know that*.” and *“I’m not sure*.” at the start of every response, whereas sometimes it fails to capture relevant facts from the knowledge base to generate adequate sentences. For example, Agent 1: *That is right! Hey do you like football?*; Agent 2: *I do. I watched the superbowl a few weeks ago, kind of boring for the big game*.; Agent 3: *Season is over!! when the season starts they only rest one weekend!*; Target Response: *Yeah, well apparently Iowa paints their locker room pink for visiting teams*; EmoKbGAN: *That is so cute. They are very That too*; EmoKb-Seq2SeqGAN: *I agree. Do you like soccer?* EmoKb-TransformerGAN: *That is a lot of football? I don’t know how much about you?* This shows that there is a large scope for improvement in modelling conversations to integrate knowledge and emotions jointly.

To demonstrate the effectiveness of the twin decoders, we compare the results obtained from the primary decoding and the secondary decoding. Table 10 shows the improvement after the second decoding. For both the cases, the secondary decoder uses more detailed knowledge than the first one.

**Table 10:** Examples of the twin decoding.

ID	Utterance	Primary decoder	Secondary decoder
1	Do you like cats?	Yeah, cats can be cool.	I love cats. cat is referred as domestic cat and wild cat.
2	Nice. Did you know U of Iowa painted the visitor locker room pink?	Yes. I have heard about it though.	In the 60’s top bowlers made twice as much money as the top football stars!

## Conclusion and future work

In this paper, we have proposed a new deep learning framework for modelling knowledge-grounded conversations using associated knowledge base and emotion labels as the guiding attributes. Building an emotion-aware conversational agent is very important to engage the users in long-time conversations and to enhance the customers’ retention. Our developed model is efficient in effectively modelling the context history, knowledge base, and target emotion label using multiple discriminators.

Overall, model performance concerning both human and automatic evaluation metric shows promising results. Analysing the generated sequences both qualitatively and quantitatively shows that the responses are highly substantial in terms of linguistic context and follow the emotional behavior up to some level. We have discussed some of the anomalous cases shown by our model in the results and analysis section. Overall, our model compared to the others generates more diverse responses as well as ensures the emotional relevancy of the responses to improve user engagement.

In future, we aim to use language models for building our discriminators. Also, we intend to fine-tune the pre-trained language models on our task for dialogue generation using knowledge selection since currently using the full knowledge, we exceed the minimum requirement of 512 dimension for training the pre-trained models.
